# Delay or Avoidance of Medical Care Because of COVID-19–Related Concerns — United States, June 2020

**DOI:** 10.15585/mmwr.mm6936a4

**Published:** 2020-09-11

**Authors:** Mark É. Czeisler, Kristy Marynak, Kristie E.N. Clarke, Zainab Salah, Iju Shakya, JoAnn M. Thierry, Nida Ali, Hannah McMillan, Joshua F. Wiley, Matthew D. Weaver, Charles A. Czeisler, Shantha M.W. Rajaratnam, Mark E. Howard

**Affiliations:** ^1^Turner Institute for Brain and Mental Health, Monash University, Melbourne, Australia; ^2^Austin Health, Melbourne, Australia; ^3^CDC COVID-19 Response Team; ^4^Johns Hopkins University Bloomberg School of Public Health, Baltimore, Maryland; ^5^Brigham and Women’s Hospital, Boston, Massachusetts; ^6^Harvard Medical School, Boston, Massachusetts; ^7^University of Melbourne, Melbourne, Australia.

Temporary disruptions in routine and nonemergency medical care access and delivery have been observed during periods of considerable community transmission of SARS-CoV-2, the virus that causes coronavirus disease 2019 (COVID-19) (*1*). However, medical care delay or avoidance might increase morbidity and mortality risk associated with treatable and preventable health conditions and might contribute to reported excess deaths directly or indirectly related to COVID-19 (*2*). To assess delay or avoidance of urgent or emergency and routine medical care because of concerns about COVID-19, a web-based survey was administered by Qualtrics, LLC, during June 24–30, 2020, to a nationwide representative sample of U.S. adults aged ≥18 years. Overall, an estimated 40.9% of U.S. adults have avoided medical care during the pandemic because of concerns about COVID-19, including 12.0% who avoided urgent or emergency care and 31.5% who avoided routine care. The estimated prevalence of urgent or emergency care avoidance was significantly higher among the following groups: unpaid caregivers for adults* versus noncaregivers (adjusted prevalence ratio [aPR] = 2.9); persons with two or more selected underlying medical conditions^†^ versus those without those conditions (aPR = 1.9); persons with health insurance versus those without health insurance (aPR = 1.8); non-Hispanic Black (Black) adults (aPR = 1.6) and Hispanic or Latino (Hispanic) adults (aPR = 1.5) versus non-Hispanic White (White) adults; young adults aged 18–24 years versus adults aged 25–44 years (aPR = 1.5); and persons with disabilities^§^ versus those without disabilities (aPR = 1.3). Given this widespread reporting of medical care avoidance because of COVID-19 concerns, especially among persons at increased risk for severe COVID-19, urgent efforts are warranted to ensure delivery of services that, if deferred, could result in patient harm. Even during the COVID-19 pandemic, persons experiencing a medical emergency should seek and be provided care without delay (*3*).

During June 24–30, 2020, a total of 5,412 (54.7%) of 9,896 eligible adults^¶^ completed web-based COVID-19 Outbreak Public Evaluation Initiative surveys administered by Qualtrics, LLC.** The Human Research Ethics Committee of Monash University (Melbourne, Australia) reviewed and approved the study protocol on human subjects research. This activity was also reviewed by CDC and was conducted consistent with applicable federal law and CDC policy.^††^ Respondents were informed of the study purposes and provided electronic consent before commencement, and investigators received anonymized responses. The 5,412 participants included 3,683 (68.1%) first-time respondents and 1,729 (31.9%) persons who had completed a related survey^§§^ during April 2–8, 2020. Among the 5,412 participants, 4,975 (91.9%) provided complete data for all variables in this analysis. Quota sampling and survey weighting^¶¶^ were employed to improve cohort representativeness of the U.S. population by gender, age, and race/ethnicity.

Respondents were asked “Have you delayed or avoided medical care due to concerns related to COVID-19?” Delay or avoidance was evaluated for emergency (e.g., care for immediate life-threatening conditions), urgent (e.g., care for immediate non–life-threatening conditions), and routine (e.g., annual check-ups) medical care. Given the potential for variation in interpretation of whether conditions were life-threatening, responses for urgent and emergency care delay or avoidance were combined for analysis. Covariates included gender; age; race/ethnicity; disability status; presence of one or more selected underlying medical conditions known to increase risk for severe COVID-19; education; essential worker status***; unpaid adult caregiver status; U.S. census region; urban/rural classification^†††^; health insurance status; whether respondents knew someone who had received a positive SARS-CoV-2 test result or had died from COVID-19; and whether the respondents believed they were at high risk for severe COVID-19. Comparisons within all these subgroups were evaluated using multivariable Poisson regression models^§§§^ with robust standard errors to estimate prevalence ratios adjusted for all covariates, 95% confidence intervals, and p-values to evaluate statistical significance (α = 0.05) using the R survey package (version 3.29) and R software (version 4.0.2; The R Foundation).

As of June 30, 2020, among 4,975 U.S. adult respondents, 40.9% reported having delayed or avoided any medical care, including urgent or emergency care (12.0%) and routine care (31.5%), because of concerns about COVID-19 (Table 1). Groups of persons among whom urgent or emergency care avoidance exceeded 20% and among whom any care avoidance exceeded 50% included adults aged 18–24 years (30.9% for urgent or emergency care; 57.2% for any care), unpaid caregivers for adults (29.8%; 64.3%), Hispanic adults (24.6%; 55.5%), persons with disabilities (22.8%; 60.3%), persons with two or more selected underlying medical conditions (22.7%; 54.7%), and students (22.7%; 50.3%). One in four unpaid caregivers reported caring for adults who were at increased risk for severe COVID-19.

**TABLE 1:** Estimated prevalence of delay or avoidance of medical care because of concerns related to COVID-19, by type of care and respondent characteristics — United States, June 30, 2020

Characteristic	No. (%)^†^	Type of medical care delayed or avoided*					
		Urgent or emergency		Routine		Any	
		%^†^	P-value^§^	%^†^	P-value^§^	%^†^	P-value^§^
**All respondents**	**4,975 (100)**	**12.0**	**—**	**31.5**	**—**	**40.9**	**—**
**Gender**							
Female	2,528 (50.8)	11.7	0.598	35.8	<0.001	44.9	<0.001
Male	2,447 (49.2)	12.3		27.0		36.7	
**Age group, yrs**							
18–24	650 (13.1)	30.9	<0.001	29.6	0.072	57.2	<0.001
25–44	1,740 (35.0)	14.9		34.2		44.8	
45–64	1,727 (34.7)	5.7		30.0		34.5	
≥65	858 (17.3)	4.4		30.3		33.5	
**Race/Ethnicity**							
White, non-Hispanic	3,168 (63.7)	6.7	<0.001	30.9	0.020	36.2	<0.001
Black, non-Hispanic	607 (12.2)	23.3		29.7		48.1	
Asian, non-Hispanic	238 (4.8)	8.6		31.3		37.7	
Other race or multiple races, non-Hispanic^¶^	150 (3.0)	15.5		23.9		37.3	
Hispanic, any race or races	813 (16.3)	24.6		36.4		55.5	
Disability**							
Yes	1,108 (22.3)	22.8	<0.001	42.9	<0.001	60.3	<0.001
No	3,867 (77.7)	8.9		28.2		35.3	
**Underlying medical condition^††^**							
No	2,537 (51.0)	8.2	<0.001	27.9	<0.001	34.7	<0.001
One	1,328 (26.7)	10.4		33.0		41.2	
Two or more	1,110 (22.3)	22.7		37.7		54.7	
**2019 household income, USD**							
<25,000	665 (13.4)	13.9	0.416	31.2	0.554	42.8	0.454
25,000–49,999	1,038 (20.9)	11.1		30.9		38.6	
50,000–99,999	1,720 (34.6)	12.5		30.5		41.1	
≥100,000	1,552 (31.2)	11.2		33.0		41.4	
**Education**							
Less than high school diploma	65 (1.3)	15.6	0.442	24.7	0.019	37.9	0.170
High school diploma	833 (16.7)	12.3		28.1		38.1	
Some college	1,302 (26.2)	13.6		29.7		40.3	
Bachelor's degree	1,755 (35.3)	11.2		34.8		43.6	
Professional degree	1,020 (20.5)	10.9		31.2		39.5	
**Employment status**							
Employed	3,049 (61.3)	14.6	<0.001	31.5	0.407	43.3	<0.001
Unemployed	630 (12.7)	8.7		34.4		39.5	
Retired	1,129 (22.7)	5.3		29.9		33.8	
Student	166 (3.3)	22.7		30.5		50.3	
**Essential worker status^§§^**							
Essential worker	1,707 (34.3)	19.5	<0.001	32.4	0.293	48.0	<0.001
Nonessential worker	1,342 (27.0)	8.4		30.3		37.3	
**Unpaid caregiver status^¶¶^**							
Unpaid caregiver for adults	1,344 (27.0)	29.8	<0.001	41.0	<0.001	64.3	<0.001
Not unpaid caregiver for adults	3,631 (73.0)	5.4		27.9		32.2	
U.S. Census region***							
Northeast	1,122 (22.6)	11.0	0.008	33.9	0.203	42.5	0.460
Midwest	936 (18.8)	8.5		32.0		38.7	
South	1,736 (34.9)	13.9		29.6		40.7	
West	1,181 (23.7)	13.0		31.5		41.5	
**Rural/Urban classification^†††^**							
Urban	4,411 (88.7)	12.3	0.103	31.5	0.763	41.2	0.216
Rural	564 (11.3)	9.4		30.9		38.2	
**Health insurance status**							
Yes	4,577 (92.0)	12.4	0.036	32.6	<0.001	42.3	<0.001
No	398 (8.0)	7.8		18.4		24.8	
**Know someone with positive test results for SARS-CoV-2^§§§^**							
Yes	989 (19.9)	8.8	0.004	40.7	<0.001	46.6	<0.001
No	3,986 (80.1)	12.8		29.2		39.5	
**Knew someone who died from COVID-19**							
Yes	364 (7.3)	10.1	0.348	41.4	<0.001	46.3	0.048
No	4,611 (92.7)	12.2		30.7		40.5	
**Believed to be in group at high risk for severe COVID-19**							
Yes	981 (19.7)	10.0	0.050	42.5	<0.001	49.4	<0.001
No	3,994 (80.3)	12.5		28.8		38.8	

**Abbreviations:** CI = confidence interval; COVID-19 = coronavirus disease 2019; USD = U.S. dollars.

* The types of medical care avoidance are not mutually exclusive; respondents had the option to indicate that they had delayed or avoided more than one type of medical care (i.e., routine medical care and urgent/emergency medical care).

^†^ Statistical raking and weight trimming were employed to improve the cross-sectional June cohort representativeness of the U.S. population by gender, age, and race/ethnicity according to the 2010 U.S. Census.

^§^ The Rao-Scott adjusted Pearson chi-squared test was used to test for differences in observed and expected frequencies among groups by characteristic for avoidance of each type of medical care (e.g., whether avoidance of routine medical care differs significantly by gender). Statistical significance was evaluated at a threshold of α = 0.05.

^¶^ “Other” race includes American Indian or Alaska Native, Native Hawaiian or Pacific Islander, or Other.

** Persons who had a disability were defined as such based on a qualifying response to either one of two questions: “Are you limited in any way in any activities because of physical, mental, or emotional condition?” and “Do you have any health conditions that require you to use special equipment, such as a cane, wheelchair, special bed, or special telephone?” https://www.cdc.gov/brfss/questionnaires/pdf-ques/2015-brfss-questionnaire-12-29-14.pdf.

^††^ Selected underlying medical conditions known to increase the risk for severe COVID-19 included in this analysis were obesity, diabetes, high blood pressure, cardiovascular disease, and any type of cancer. Obesity is defined as body mass index ≥30 kg/m^2^ and was calculated from self-reported height and weight (https://www.cdc.gov/healthyweight/assessing/bmi/adult_bmi/index.html). The remaining conditions were assessed using the question “Have you ever been diagnosed with any of the following conditions?” with response options of 1) “Never”; 2) “Yes, I have in the past, but don’t have it now”; 3) “Yes I have, but I do not regularly take medications or receive treatment”; and 4) “Yes I have, and I am regularly taking medications or receiving treatment.” Respondents who answered that they have been diagnosed and chose either response 3 or 4 were considered as having the specified medical condition.

^§§^ Essential worker status was self-reported.

^¶¶^ Unpaid caregiver status was self-reported. Unpaid caregivers for adults were defined as having provided unpaid care to a relative or friend aged ≥18 years at any time in the last 3 months. Examples provided to survey respondents included helping with personal needs, household chores, health care tasks, managing a person’s finances, taking them to a doctor’s appointment, arranging for outside services, and visiting regularly to see how they are doing.

*** Region classification was determined by using the U.S. Census Bureau’s Census Regions and Divisions. https://www2.census.gov/geo/pdfs/maps-data/maps/reference/us_regdiv.pdf.

^†††^ Rural-urban classification was determined by using self-reported ZIP codes according to the Federal Office of Rural Health Policy definition of rurality. https://www.hrsa.gov/rural-health/about-us/definition/datafiles.html.

^§§§^ For this question, respondents were asked to select the following statement, if applicable: “I know someone who has tested positive for COVID-19.”

In the multivariable Poisson regression models, differences within groups were observed for urgent or emergency care avoidance (Figure) and any care avoidance (Table 2). Adjusted prevalence of urgent or emergency care avoidance was significantly higher among unpaid caregivers for adults versus noncaregivers (2.9; 2.3–3.6); persons with two or more selected underlying medical conditions versus those without those conditions (1.9; 1.5–2.4); persons with health insurance versus those without health insurance (1.8; 1.2–2.8); Black adults (1.6; 1.3–2.1) and Hispanic adults (1.5; 1.2–2.0) versus White adults; young adults aged 18–24 years versus adults aged 25–44 years (1.5; 1.2–1.8); and persons with disabilities versus those without disabilities (1.3; 1.1–1.5). Avoidance of urgent or emergency care was significantly lower among adults aged ≥45 years than among younger adults.

**FIGURE:**
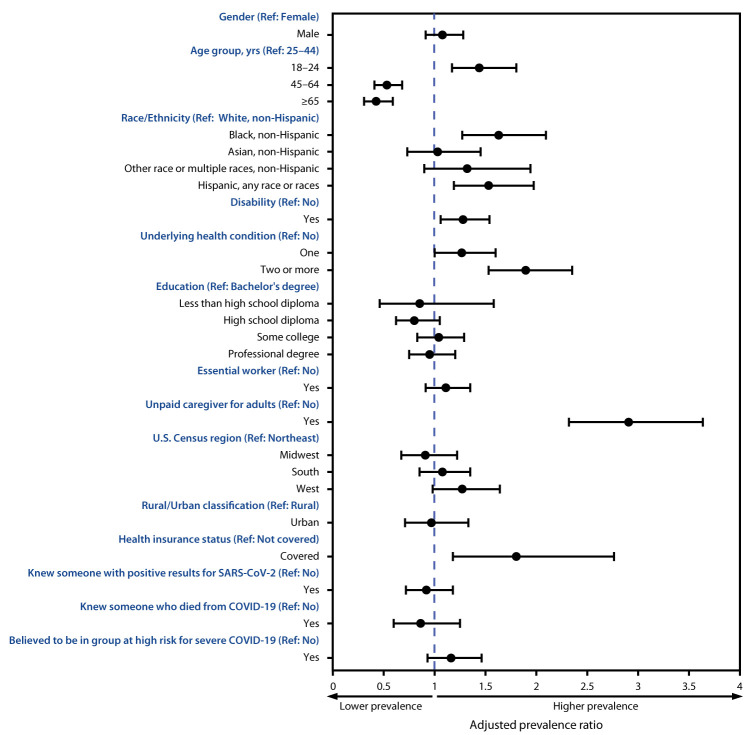
Adjusted prevalence ratios*^,^^†^ for characteristics**^§^****^,^****^¶^**^,^**^,^^††^ associated with delay or avoidance of urgent or emergency medical care because of concerns related to COVID-19 — United States, June 30, 2020 **Abbreviation:** COVID-19 = coronavirus disease 2019. * Comparisons within subgroups were evaluated using Poisson regressions used to calculate a prevalence ratio adjusted for all characteristics shown in figure. ^†^ 95% confidence intervals indicated with error bars. ^§^ “Other” race includes American Indian or Alaska Native, Native Hawaiian or Pacific Islander, or Other. ^¶^ Selected underlying medical conditions known to increase the risk for severe COVID-19 were obesity, diabetes, high blood pressure, cardiovascular disease, and any type of cancer. Obesity is defined as body mass index ≥30 kg/m^2^ and was calculated from self-reported height and weight (https://www.cdc.gov/healthyweight/assessing/bmi/adult_bmi/index.html). The remaining conditions were assessed using the question “Have you ever been diagnosed with any of the following conditions?” with response options of 1) “Never”; 2) “Yes, I have in the past, but don’t have it now”; 3) “Yes I have, but I do not regularly take medications or receive treatment”; and 4) “Yes I have, and I am regularly taking medications or receiving treatment.” Respondents who answered that they have been diagnosed and chose either response 3 or 4 were considered as having the specified medical condition. ** Essential worker status was self-reported. For the adjusted prevalence ratios, essential workers were compared with all other respondents (including those who were nonessential workers, retired, unemployed, and students). ^††^ Unpaid caregiver status was self-reported. Unpaid caregivers for adults were defined as having provided unpaid care to a relative or friend aged ≥18 years to help them take care of themselves at any time in the last 3 months.

**TABLE 2:** Characteristics associated with delay or avoidance of any medical care because of concerns related to COVID-19 — United States, June 30, 2020

Characteristic	Weighted* no.	Avoided or delayed any medical care		
		aPR^†^	(95% CI^†^)	P-value^†^
**All respondents**	**4,975**	**—**	**—**	**—**
**Gender**				
Female	2,528	Referent	—	—
Male	2,447	0.81	(0.75–0.87)^§^	<0.001
**Age group, yrs**				
18–24	650	1.12	(1.01–1.25)^§^	0.035
25–44	1,740	Referent	—	—
45–64	1,727	0.80	(0.72–0.88)^§^	<0.001
≥65	858	0.72	(0.64–0.81)^§^	<0.001
**Race/Ethnicity**				
White, non-Hispanic	3,168	Referent	—	—
Black, non-Hispanic	607	1.07	(0.96–1.19)	0.235
Asian, non-Hispanic	238	1.04	(0.91–1.18)	0.567
Other race or multiple races, non-Hispanic^¶^	150	0.87	(0.71–1.07)	0.196
Hispanic, any race or races	813	1.15	(1.03–1.27)^§^	0.012
Disability**				
Yes	1,108	1.33	(1.23–1.43)^§^	<0.001
No	3,867	Referent	—	—
**Underlying medical condition^††^**				
No	2,537	Referent	—	—
One	1,328	1.15	(1.05–1.25)^§^	0.004
Two or more	1,110	1.31	(1.20–1.42)^§^	<0.001
**Education**				
Less than high school diploma	65	0.72	(0.53–0.98)^§^	0.037
High school diploma	833	0.79	(0.71–0.89)^§^	<0.001
Some college	1,302	0.85	(0.78–0.93)^§^	0.001
Bachelor's degree	1,755	Referent	—	—
Professional degree	1,020	0.90	(0.82–0.98)^§^	0.019
**Essential workers vs others^§§^**				
Essential workers	1,707	1.00	(0.92–1.09)	0.960
Other respondents (nonessential workers, retired persons, unemployed persons, and students)	3,268	Referent	—	—
**Unpaid caregiver status^¶¶^**				
Unpaid caregiver for adults	1,344	1.64	(1.52–1.78)^§^	<0.001
Not unpaid caregiver for adults	3,631	Referent	—	—
U.S. Census region***				
Northeast	1,122	Referent	—	—
Midwest	936	0.93	(0.83–1.04)	0.214
South	1,736	0.90	(0.82–0.99)^§^	0.028
West	1,181	0.99	(0.89–1.09)	0.808
**Rural/Urban classification^†††^**				
Urban	4,411	1.00	(0.89–1.12)	0.993
Rural	564	Referent	—	—
**Health insurance status**				
Yes	4,577	1.61	(1.31–1.98)^§^	<0.001
No	398	Referent	—	—
**Know someone with positive test results for SARS-CoV-2^§§§^**				
Yes	989	1.22	(1.12–1.33)^§^	<0.001
No	3,986	Referent	—	—
**Knew someone who died from COVID-19**				
Yes	364	0.99	(0.88–1.12)	0.860
No	4,611	Referent	—	—
**Believed to be in a group at high risk for severe COVID-19**				
Yes	981	1.33	(1.23–1.44)^§^	<0.001
No	3,994	Referent	—	—

**Abbreviations:** aPR = adjusted prevalence ratio; CI = confidence interval; COVID-19 = coronavirus disease 2019.

* Statistical raking and weight trimming were employed to improve the cross-sectional June cohort representativeness of the U.S. population by gender, age, and race/ethnicity according to the 2010 U.S. Census.

^†^ Comparisons within subgroups were evaluated using Poisson regressions used to calculate a prevalence ratio adjusted for all characteristics listed, as well as a 95% CI and p-value. Statistical significance was evaluated at a threshold of α = 0.05.

^§^ P-value calculated using Poisson regression among respondents within a characteristic is statistically significant at levels of p<0.05.

^¶^ “Other” race includes American Indian or Alaska Native, Native Hawaiian or Pacific Islander, or Other.

** Persons who had a disability were defined based on a qualifying response to either one of two questions: “Are you limited in any way in any activities because of physical, mental, or emotional condition?” and “Do you have any health conditions that require you to use special equipment, such as a cane, wheelchair, special bed, or special telephone?” https://www.cdc.gov/brfss/questionnaires/pdf-ques/2015-brfss-questionnaire-12-29-14.pdf.

^††^ Selected underlying medical conditions known to increase the risk for severe COVID-19 were obesity, diabetes, high blood pressure, cardiovascular disease, and any type of cancer. Obesity is defined as body mass index ≥30 kg/m^2^ and was calculated from self-reported height and weight (https://www.cdc.gov/healthyweight/assessing/bmi/adult_bmi/index.html). The remaining conditions were assessed using the question “Have you ever been diagnosed with any of the following conditions?” with response options of 1) “Never”; 2) “Yes, I have in the past, but don’t have it now”; 3) “Yes I have, but I do not regularly take medications or receive treatment”; and 4) “Yes I have, and I am regularly taking medications or receiving treatment.” Respondents who answered that they have been diagnosed and chose either response 3 or 4 were considered as having the specified medical condition.

^§§^ Essential worker status was self-reported. For the adjusted prevalence ratios, essential workers were compared with all other respondents (including those who were nonessential workers, retired, unemployed, and students).

^¶¶^ Unpaid caregiver status was self-reported. Unpaid caregivers for adults were defined as having provided unpaid care to a relative or friend aged ≥18 years at any time in the last 3 months. Examples provided to survey respondents included helping with personal needs, household chores, health care tasks, managing a person’s finances, taking them to a doctor’s appointment, arranging for outside services, and visiting regularly to see how they are doing.

*** Region classification was determined by using the U.S. Census Bureau’s Census Regions and Divisions. https://www2.census.gov/geo/pdfs/maps-data/maps/reference/us_regdiv.pdf.

^†††^ Rural/urban classification was determined by using self-reported ZIP codes according to the Federal Office of Rural Health Policy definition of rurality. https://www.hrsa.gov/rural-health/about-us/definition/datafiles.html.

^§§§^ For this question, respondents were asked to select the following statement, if applicable: “I know someone who has tested positive for COVID-19.”

## Discussion

As of June 30, 2020, an estimated 41% of U.S. adults reported having delayed or avoided medical care during the pandemic because of concerns about COVID-19, including 12% who reported having avoided urgent or emergency care. These findings align with recent reports that hospital admissions, overall emergency department (ED) visits, and the number of ED visits for heart attack, stroke, and hyperglycemic crisis have declined since the start of the pandemic (*3*–*5*), and that excess deaths directly or indirectly related to COVID-19 have increased in 2020 versus prior years (*2*). Nearly one third of adult respondents reported having delayed or avoided routine medical care, which might reflect adherence to community mitigation efforts such as stay-at-home orders, temporary closures of health facilities, or additional factors. However, if routine care avoidance were to be sustained, adults could miss opportunities for management of chronic conditions, receipt of routine vaccinations, or early detection of new conditions, which might worsen outcomes.

Avoidance of both urgent or emergency and routine medical care because of COVID-19 concerns was highly prevalent among unpaid caregivers for adults, respondents with two or more underlying medical conditions, and persons with disabilities. For caregivers who reported caring for adults at increased risk for severe COVID-19, concern about exposure of care recipients might contribute to care avoidance. Persons with underlying medical conditions that increase their risk for severe COVID-19 (*6*) are more likely to require care to monitor and treat these conditions, potentially contributing to their more frequent report of avoidance. Moreover, persons at increased risk for severe COVID-19 might have avoided health care facilities because of perceived or actual increased risk of exposure to SARS-CoV-2, particularly at the onset of the pandemic. However, health care facilities are implementing important safety precautions to reduce the risk of SARS-CoV-2 infection among patients and personnel. In contrast, delay or avoidance of care might increase risk for life-threatening medical emergencies. In a recent study, states with large numbers of COVID-19–associated deaths also experienced large proportional increases in deaths from other underlying causes, including diabetes and cardiovascular disease (*7*). For persons with disabilities, accessing medical services might be challenging because of disruptions in essential support services, which can result in adverse health outcomes. Medical services for persons with disabilities might also be disrupted because of reduced availability of accessible transportation, reduced communication in accessible formats, perceptions of SARS-CoV-2 exposure risk, and specialized needs that are difficult to address with routine telehealth delivery during the pandemic response. Increasing accessibility of medical and telehealth services^¶¶¶^ might help prevent delay of needed care.

Increased prevalences of reported urgent or emergency care avoidance among Black adults and Hispanic adults compared with White adults are especially concerning given increased COVID-19-associated mortality among Black adults and Hispanic adults (*8*). In the United States, the age-adjusted COVID-19 hospitalization rates are approximately five times higher among Black persons and four times higher among Hispanic persons than are those among White persons (*9*). Factors contributing to racial and ethnic disparities in SARS-CoV-2 exposure, illness, and mortality might include long-standing structural inequities that influence life expectancy, including prevalence and underlying medical conditions, health insurance status, and health care access and utilization, as well as work and living circumstances, including use of public transportation and essential worker status. Communities, health care systems, and public health agencies can foster equity by working together to ensure access to information, testing, and care to assure maintenance and management of physical and mental health.

The higher prevalence of medical care delay or avoidance among respondents with health insurance versus those without insurance might reflect differences in medical care-seeking behaviors. Before the pandemic, persons without insurance sought medical care much less frequently than did those with insurance (*10*), resulting in fewer opportunities for medical care delay or avoidance.

The findings in this report are subject to at least five limitations. First, self-reported data are subject to recall, response, and social desirability biases. Second, the survey did not assess reasons for COVID-19–associated care avoidance, such as adherence to public health recommendations; closure of health care provider facilities; reduced availability of public transportation; fear of exposure to infection with SARS-CoV-2; or availability, accessibility, and acceptance or recognition of telemedicine as a means of providing care in lieu of in-person services. Third, the survey did not assess baseline patterns of care-seeking or timing or duration of care avoidance. Fourth, perceptions of whether a condition was life-threatening might vary among respondents. Finally, although quota sampling methods and survey weighting were employed to improve cohort representativeness, this web-based survey might not be fully representative of the U.S. population for income, educational attainment, and access to technology. However, the findings are consistent with reported declines in hospital admissions and ED visits during the pandemic (*3*–*5*).

CDC has issued guidance to assist persons at increased risk for severe COVID-19 in staying healthy and safely following treatment plans**** and to prepare health care facilities to safely deliver care during the pandemic.^††††^ Additional public outreach in accessible formats tailored for diverse audiences might encourage these persons to seek necessary care. Messages could highlight the risks of delaying needed care, especially among persons with underlying medical conditions, and the importance of timely emergency care. Patient concerns related to potential exposure to SARS-CoV-2 in health care settings could be addressed by describing facilities’ precautions to reduce exposure risk.

Further exploration of underlying reasons for medical care avoidance is needed, including among persons with disabilities, persons with underlying health conditions, unpaid caregivers for adults, and those who face structural inequities. If care were avoided because of concern about SARS-CoV-2 exposure or if there were closures or limited options for in-person services, providing accessible telehealth or in-home health care could address some care needs. Even during the COVID-19 pandemic, persons experiencing a medical emergency should seek and be provided care without delay (*3*).

**Summary**

**What is already known about this topic?**

Delayed or avoided medical care might increase morbidity and mortality associated with both chronic and acute health conditions.

**What is added by this report?**

By June 30, 2020, because of concerns about COVID-19, an estimated 41% of U.S. adults had delayed or avoided medical care including urgent or emergency care (12%) and routine care (32%). Avoidance of urgent or emergency care was more prevalent among unpaid caregivers for adults, persons with underlying medical conditions, Black adults, Hispanic adults, young adults, and persons with disabilities.

**What are the implications for public health practice?**

Understanding factors associated with medical care avoidance can inform targeted care delivery approaches and communication efforts encouraging persons to safely seek timely routine, urgent, and emergency care.

## Footnotes

* Unpaid caregiver status was self-reported. The definition of an unpaid caregiver for adults was having provided unpaid care to a relative or friend aged ≥18 years to help them take care of themselves at any time in the last 3 months. Examples provided to survey respondents included helping with personal needs, household chores, health care tasks, managing a person’s finances, taking them to a doctor’s appointment, arranging for outside services, and visiting regularly to see how they are doing.

† Selected underlying medical conditions known to increase the risk for severe COVID-19 included in this analysis were obesity (body mass index [BMI] ≥30 kg/m^2^), diabetes, high blood pressure, cardiovascular disease, and any type of cancer. BMI was calculated from self-reported height and weight as BMI = weight (lb)/[height (in)]^2^ x 703 (https://www.cdc.gov/healthyweight/assessing/bmi/adult_bmi/index.html). The remaining conditions were assessed using the following question: “Have you ever been diagnosed with any of the following conditions?” with the following four response options: 1) “Never”; 2) “Yes, I have in the past, but don’t have it now”; 3) “Yes I have, but I do not regularly take medications or receive treatment”; and 4) “Yes I have, and I am regularly taking medications or receiving treatment.” Respondents who answered that they have been diagnosed and chose either response 3 or 4 were considered as having the specified medical condition.

§ Persons who had a disability were defined as such based on a qualifying response to either one of two questions: “Are you limited in any way in any activities because of physical, mental, or emotional condition?” and “Do you have any health conditions that require you to use special equipment, such as a cane, wheelchair, special bed, or special telephone?” https://www.cdc.gov/brfss/questionnaires/pdf-ques/2015-brfss-questionnaire-12-29-14.pdf.

¶ Eligibility to complete a survey during June 24–30, 2020, was determined following electronic contact of potential participants based on a minimum age of 18 years and residence within the United States. Age and residence were assessed using screening questions without indication of eligibility criteria before commencement of the earliest survey (recontacted respondents: April 2–8, 2020; first-time respondents: June 24–30, 2020). Residence was reassessed among recontacted respondents during June 24–30, and one respondent whose primary residence had changed to outside of the United States was excluded from the analysis. Country-specific geolocation verification via IP address mapping was used to ensure respondents were responding from the United States. Informed consent was obtained electronically during June 24–30, 2020, before enrollment into the study as a participant. All surveys underwent Qualtrics, LLC data quality screening procedures, including algorithmic and keystroke analysis for attention patterns, click-through behavior, duplicate responses, machine responses, and inattentiveness. Respondents who failed an attention or speed check, along with any responses that failed data quality screening procedures, were excluded from the analysis (6.6%).

** The COVID-19 Outbreak Public Evaluation (COPE) Initiative (www.thecopeinitiative.org) is designed to assess public attitudes, behaviors, and beliefs related to the coronavirus disease 2019 (COVID-19) pandemic, and to evaluate the mental and physical health consequences of the pandemic. The COPE Initiative surveys included in this analysis were administered by Qualtrics, LLC (https://www.qualtrics.com/), a commercial survey company with a network of participant pools comprising hundreds of suppliers and with varying recruitment methodologies that include digital advertisements and promotions, word-of-mouth and membership referrals, social networks, television and radio advertisements, and offline mail-based approaches.

†† 45 C.F.R. part 46, 21 C.F.R. part 56; 42 U.S.C. Sect. 241(d); 5 U.S.C. Sect. 552a; 44 U.S.C. Sect. 3501 et seq.

§§ https://www.medrxiv.org/content/10.1101/2020.04.22.20076141v1.

¶¶ Statistical raking and weight trimming were employed to improve the cross-sectional June cohort representativeness of the U.S. population by gender, age, and race/ethnicity according to the 2010 U.S. Census.

*** Essential worker status was self-reported. For the aPRs, essential workers were compared with all other respondents (including those who were nonessential workers, retired, unemployed, and students).

††† Rural-urban classification was determined by using self-reported ZIP codes according to the Federal Office of Rural Health Policy definition of rurality. https://www.hrsa.gov/rural-health/about-us/definition/datafiles.html.

§§§ Reference groups were chosen for ease of interpretation. For example, the household income level of $50,000–$99,999 was selected as the reference group because the median household income was $61,937 in the United States in 2018. https://www.census.gov/content/dam/Census/library/publications/2019/acs/acsbr18-01.pdf.

¶¶¶ https://www.cdc.gov/coronavirus/2019-ncov/hcp/telehealth.html.

**** https://www.cdc.gov/coronavirus/2019-ncov/need-extra-precautions/people-with-medical-conditions.html.

†††† https://www.cdc.gov/coronavirus/2019-ncov/hcp/us-healthcare-facilities.html.
